# Does Gender Impact Intensity of Care Provided to Older Medical Intensive Care Unit Patients?

**DOI:** 10.1155/2010/404608

**Published:** 2010-10-20

**Authors:** Kathleen M. Akgün, Terrence E. Murphy, Katy L. B. Araujo, Peter H. Van Ness, Margaret Pisani

**Affiliations:** ^1^Pulmonary & Critical Care Section, Department of Internal Medicine, Yale University School of Medicine, 333 Cedar Street P.O. Box 208057, New Haven, CT 06520-8057, USA; ^2^Geriatrics Section, and the Program on Aging, Department of Internal Medicine, Yale University School of Medicine, New Haven, CT 06520-8057, USA; ^3^Division of Chronic Disease Epidemiology, Yale School of Public Health, New Haven, CT 06520-8057, USA

## Abstract

*Introduction*. Women receive less aggressive critical care than men based on prior studies. No documented studies evaluate whether men and women are treated equally in the medical intensive care unit (MICU). The Therapeutic Intervention Scoring System-28 (TISS-28) has been used to examine gender differences in mixed ICU studies. However, it has not been used to evaluate equivalence of care in older MICU patients. We hypothesize that given nonsignificant, baseline health differences between genders at MICU admission, the level of care provided would be equivalent. *Methods*. Prospective cohort of 309 patients ≥60 years old in the MICU of an urban university teaching hospital. Explanatory variables were demographic data and baseline measures. Primary outcomes were TISS-28 scores and MICU interventions. We compare TISS-28 scores by gender using a statistical test of equivalence. *Results*. Women were older and had more chronic respiratory failure at MICU admission. Using equivalence limits of ±15% on gender-based scores of TISS-28, MICU interventions were equivalent. Supplementary analysis showed no statistically significant association between gender and mortality. *Conclusions*. In contrast with other reports from the cardiac critical care literature, as measured by the TISS-28, gender-based care delivered to older MICU patients in this cohort was equivalent.

## 1. Introduction

Currently 12.6% of the total population in the U.S. is 65 years of age or older [1]. This number is expected to increase to 16% by 2020. Within the 65 and older age group, 58% are women [1]. As the population ages, medical intensive care unit (MICU) utilization is likely to increase. Prior studies investigating aging and critical illness show that MICU outcomes are primarily determined by severity of illness and respiratory failure rather than age [2–4]. 

Initial studies examining gender differences in the ICU were conducted with cardiac patients. These studies demonstrated that women were older and sicker at the time of angioplasty or surgical bypass, receive fewer invasive procedures, such as implantable cardioverter defibrillators and heart transplants, and were less likely to receive appropriate medications during and after non-ST segment elevation myocardial infarctions [5]. These findings suggest potential treatment disparities between men and women. However, the importance of gender for MICU outcomes in older patients has not been fully established [2–4, 6, 7]. 

Severity of illness scores such as the Acute Physiology and Chronic Health Evaluation (APACHE II), Sequential Organ Failure Assessment (SOFA), and the Simplified Acute Physiology Score (SAPS II) are useful descriptors for patient acuity and predictors of mortality but do not account for the patient care-related workload required for ICU patients. The Therapeutic Intervention Scoring System-28 (TISS-28) is a simple, reliable tool for describing both the severity of illness and the intensity of nursing care delivered to ICU patients [8, 9]. The TISS-28 has been used extensively in older ICU populations, exhibiting negative correlation with age, particularly in patients older than 85 years [10]. 

Whether there are gender differences in intensity of care delivered to older MICU patients using the TISS-28 has not been investigated. This study sought to determine whether there were differences in provision of MICU care and outcomes according to gender in an older cohort of patients. We also examined factors at MICU admission and during the course of the MICU stay according to gender. We hypothesized that if there were minimal or no differences in baseline health status of men and women upon MICU admission, then care provided to men and women in our MICU would be equivalent. This study is novel in using a validated scoring system (TISS-28) to formally test for statistical equivalence of MICU care between older men and women.

## 2. Materials and Methods

### 2.1. Participants and Setting

 The study was a prospective cohort of 309 patients aged 60 years and older in the medical intensive care unit of Yale-New Haven Hospital (YNHH), who were enrolled from September 5, 2002 to September 30, 2004. YNHH is a 900-bed urban teaching hospital with a 14-bed medical ICU (MICU) serving a large urban community as well as a referral population. Patients were excluded if there was no identifiable proxy to provide information about the patient, if they were transferred from another hospital or ICU, or if the patients died before the proxy was identified. Informed consent for participation was obtained from the proxy and patients according to procedures approved by the Institutional Review Board of Yale University School of Medicine.

### 2.2. Study Procedures

#### 2.2.1. Proxy Interviews

As previously described, proxy respondents were the primary sources of baseline information due to critical illness [11–14]. Information on premorbid functional and cognitive status was obtained.

#### 2.2.2. Patient Factors

 Charts were reviewed at the time of enrollment to obtain demographic information, admission diagnosis, preexisting depression, medication use at admission, and the Charlson Comorbidity Index (CCI) [15]. Severity of illness was assessed with the Acute Physiology and Chronic Health Evaluation II Score (APACHE II) [16]. 

#### 2.2.3. ICU and Hospital Factors

Use of MICU interventions such as mechanical ventilation, vasopressors, new onset dialysis, and pulmonary artery catheterization were recorded. Total number of days in the MICU and requiring mechanical ventilation were recorded to calculate median values. The full range of days in the MICU or on mechanical ventilation for the entire cohort was measured. Patients were assessed daily for delirium. Changes in resuscitation status during the MICU admission were documented. Continuous sedative and narcotic drug administration were tracked using Infusion Administration Pumps which record total dose delivered to the patient.

#### 2.2.4. TISS-28

The TISS-28 is a simple, validated scoring system designed to quantify severity of patient illness and therapeutic activities in the ICU in terms of nursing workload [9]. TISS-28 scores range from 1 to 78 points. Each TISS-28 point correlates with 10.6 minutes of nursing care [9]. The TISS-28 score is compiled from seven general groups: Basic Activities (dressing changes, laboratory blood draws, single medication administration, etc.), Ventilatory Support (mechanical ventilation, care of airway, intratracheal suctioning, etc.), Cardiovascular Support (arterial lines, central venous lines, vasoactive medications, intravenous fluid resuscitation, etc.), Renal Support (active diuresis, quantitative urine output, dialysis), Neurologic Support (measurement of intracranial pressures), Metabolic Support (treatment of complicated metabolic acidosis or alkalosis, enteral feeding, intravenous hyperalimentation), and Specific Interventions (pacemakers, cardioversions, endoscopies, emergencies surgeries, etc.). 

For this study, the information for the TISS-28 was collected prospectively from the nurses' bedside flow sheet and the medical record. Data from the entire MICU stay, ranging from admission through discharge, were utilized in the calculation. For patients who died during their MICU stay, the TISS-28 was calculated to include all interventions through time of death.

### 2.3. Outcomes

We examined gender differences at three time points. We compared baseline factors at MICU admission, interventions during the MICU stay including the TISS-28 scores and outcomes including mortality and length of stay. Fifteen-month mortality was determined by follow-up telephone interview.

### 2.4. Statistical Analysis

Admission characteristics stratified by gender were summarized with means and standard deviations or medians and interquartile ranges for continuous variables, and with counts and percentages for dichotomous variables. Tests for baseline gender differences were made using *t*-tests or Wilcoxon rank sum tests for continuous variables and Pearson chi-square or Fisher's exact test for dichotomous variables. For these tests the null hypotheses assumed equality of admission characteristics for men and women.

When comparing two groups, a lack of statistical difference is a different conclusion than saying that two groups received the same treatment, the latter a much less stringent test. We wanted to evaluate if treatment between men and women was the same using a formal test of statistical equivalence. A nonparametric, Mann-Whitney test of equivalence for discrete distributions was used to compare the TISS-28 scores between men and women during their stay in the MICU. The null hypothesis in this case was that men and women received different amounts of overall critical care. We chose equivalence limits of ±15% to reflect the percentile differences around the median value on the TISS-28 scale that we regard as being clinically equivalent based on clinical experience with critical care for older persons in the MICU. These equivalence limits correspond to an approximate change of ±5 points between the median TISS values of the gender subgroups. Because 4 points are assigned to intubation, a change of 5 points at the group level would reflect even larger treatment differences “on average” between individual men and women.

The equivalence test was performed using the SAS macro “mwtie_xy” written by Wellek [17] with *P* ≤ .05 indicating statistical significance. In supplementary analysis we examined time to death within 15 months of MICU admission with a Cox proportional hazards multivariable model [18] that included covariates for age, APACHE II at admission, gender, TISS-28, and the interaction between gender and TISS-28. These covariates were chosen based on prior ICU studies reporting that mortality is most closely tied to APACHE II scores. Given that the TISS-28 and the interaction between the TISS-28 and gender were the primary outcomes of interest and that the TISS-28 correlates with risk of death, these were additional covariates chosen. All statistical tests were two tailed, with *P* < .05 indicating significance. Analysis was performed with SAS version 9.1.3 software [19, 20].

## 3. Results 

### 3.1. Comparisons between Men and Women Admitted to the MICU

In this older MICU cohort, women were older than men (75.6 years versus 73.7 years; *P* = .046), were more frequently enrolled in Medicaid (18% versus 9%;  *P* = .02), and had more preexisting chronic respiratory failure (39% versus 25%;  *P* = .01). More men were admitted with gastrointestinal hemorrhage than women (23% versus 12%, *P* = .008). Women were more likely than men to have a diagnosis of depression and to be taking antidepressants prior to admission to the ICU (30% versus 19%;  *P* = .03). However, there were no significant differences between men and women regarding APACHE II score on MICU admission or “Do Not Resuscitate (DNR)” or “Do Not Intubate (DNI)” orders. Additional results regarding admission characteristics for men and women enrolled are included in Table 1. There were also no statistical differences in ICU interventions such as intubation, days of intubation, or ICU length of stay between men and women (Table 2).

### 3.2. TISS-28 Scores, APACHE II Scores and MICU Mortality

The amount of critical care delivered to women was equivalent to men based on their respective TISS-28 scores. The statistical test of equivalence employed equivalence limits of ±15 percent around the cohort's median value of TISS-28 scores. Additionally, there was no evidence of statistically significant differences between men and women in rates of specific MICU interventions such as intubation, tracheostomy, renal replacement therapy, or change in code status to “Less aggressive”. Table 2 compares selected specific components of the TISS-28 scores related to patients in the MICU by gender. TISS-28 scores significantly correlated with APACHE II scores (Kendall Tau B 0.27311  (<0.0001) and risk of death in the MICU (Kendall Tau B 0.26377 (<0.0001). There was no evidence of gender-based differences in medication administration, or MICU or hospital length of stay. 

We also examined TISS-28 scores by age category. The median (range) TISS-28 scores for patients age 60–69 (*n* = 87) was 27 (9–50), for patients age 70–79 (*n* = 124) was 25 (9–43) and for patients age 80 (*n* = 98) and older was 23 (9–46).

### 3.3. Patient Discharge and Fifteen-Month Mortality

In supplementary analysis women were less likely to be discharged home (42% versus 52%; *P* = .13) although the difference was not statistically significant. There was no association between gender and mortality up to 15 months after ICU admission. There was also no association between 15-month mortality and the interaction of gender and TISS score (Table 3).

## 4. Discussion

While gender differences in ICU care delivered have been reported in the cardiac critical care literature and some mixed medical and surgical ICUs, we did not detect any evidence of gender-based differences in rates of MICU interventions when looking at specific aspects of critical care. In addition, there was no evidence of statistically significant gender-based differences in administration of medications in the MICU. After controlling for age and severity of illness, there was no evidence of a statistically significant difference in ICU or 15-month mortality between men and women.

Men admitted to the MICU were younger than women, likely related to increased longevity in women in the general population. Additional differences between men and women on MICU admission included receipt of Medicaid, chronic respiratory failure and admission diagnosis of gastrointestinal bleed. Given these minimal differences between men and women on MICU admission, our conditional hypothesis that there would be equivalent care delivered to men and women if there were minimal baseline differences was supported by these study results. 

Our study showed that women were more likely to have underlying chronic pulmonary disease compared to men. With increasing rates of women smoking, there has been a shift towards more women than men dying from chronic obstructive pulmonary disease (COPD) [21]. Men were more likely to be admitted with gastrointestinal hemorrhage in our cohort. This finding conforms to data that demonstrates male gender is associated with increased risk factors for gastrointestinal hemorrhage, including peptic ulcer disease, colonic polyps, or gastrointestinal malignancies [22–25].

In our study, the average TISS-28 score in our older MICU population was 25 for men and 26 for women which is consistent with the range of TISS-28 scores from other studies. In a study of mixed medical and surgical ICUs in Brazil, the average TISS-28 for all patients who stayed in the ICU for at least 24 hours was 23 with a range from 14–32 [26]. The majority of these patients were admitted to surgical ICUs. Mean TISS-28 scores in prior studies of predominantly medical ICUs range between 22–33.8 [27–29]. TISS-28 scores and in-hospital mortality have shown a positive correlation in patients older than 65 years of age [30]; our study also found a positive correlation between TISS-28 scores and APACHE II scores (Kendall Tau B 0.27311 (<0.0001) and mortality (ICU, Kendall Tau B: 0.26377 (<0.0001). Older patients may be more vulnerable to poor ICU outcomes if they receive less aggressive care despite a greater severity of illness [31]. 

The current study shows that the care delivered to older men and women as measured by the TISS-28 was equivalent in our MICU. Treatment disparities between men and women have been observed in other studies of critically ill patients and may impact outcomes according to level of aggressive care [5, 29, 32]. A large Belgian study of mixed medical and surgical ICU patients showed that women had a higher mortality than men, particularly women greater than 50 years old during their first days of ICU admission [33]; however, this study did not adjust for severity of illness. Another study examining gender difference in acute myocardial infarction found that while there were no differences in diagnostic or treatment procedures between men and women, female sex was an independent predictor for adverse events including reinfarction, death, in-patient stroke, and postinfarction angina [34]. 

Conversely, in a single-site study of a surgical ICU by Wichmann et al. where less women were referred for care in the ICU, there was no difference in mortality between men and women who were admitted to the surgical ICU [35]. A large Finnish study of surgical and medical ICUs found that men had a modest but significantly higher TISS-28 score [29]. Despite this, older men had an increased risk of death after adjusting for severity of illness. In the medical subgroup, there was no difference in mortality between men and women [29]. In another study of critically ill medical and surgical patients from Austria, men received more invasive procedures such as mechanical ventilation, renal replacement therapy, and vasoactive medications than women although women had a greater severity of illness [32]. These differences were observed in all age groups but were most pronounced in the older population (age 61–80 years). Even with the observed differences in level of care between men and women, there were no differences in outcomes or mortality between men and women in multivariate analysis [32]. 

While studies show equivalent or greater intensive care administered to men, other data suggests that neurohumoral changes in men may have greater adverse effects on their survival after critical illness. Testosterone has immunosuppressive characteristics with detrimental effects after trauma-hemorrhage injury in animal models, increasing susceptibility to infections or sepsis [35–37]. Men with severe infections have elevated 17*β*-estradiol and progesterone levels which are associated with increased mortality [38]. Similarly, women with severe infections had higher levels of 17*β*-estradiol and testosterone which were also associated with higher mortality [38]. In addition to male and female sex hormones, biomarkers such as tumor necrosis factor, interleukin-10, and interleukin-6 are postulated to play a role in these gender disparities. 

The main limitation of this study is that it was performed in a single ICU in a large academic medical center so results may not be representative of care provided in other subspecialty ICUs or in community hospitals. Another limitation of this study is that the TISS-28 does not account for patient preferences and acceptable levels of intensive care. In addition, the TISS scoring systems were derived to measure ICU nursing activities which may not fully correlate with aggressiveness of ICU care. Strengths include the large sample size of older ICU patients with equal numbers of men and woman along with the high participation. In addition, we collected a detailed, clinically rich prospective data set using validated instruments.

In summary, this study is novel in demonstrating that treatment of men and women in this ICU cohort is not only not different but is statistically equivalent using equivalence limits based on clinical experience in caring for older MICU patients. Finally, there was no difference in mortality between men and women in this cohort.

## 5. Conclusions

In contrast with prior studies, men and women in this older MICU cohort with similar baseline characteristics on MICU admission received equivalent critical care based on their TISS-28 scores. In this study, MICU and 15-month mortality were also similar between men and women. As the U.S. population ages and medical technology advances, there will be greater need and opportunity for interventions in medical ICUs. It will be valuable to use reliable measurements for the amount of care provided and weigh their impact on ICU survival. While our study did not show significant differences in MICU care or 15-month mortality between men and women, gender-based treatments and mortality should continue to be examined for biological differences between men and women, patient and cultural influences, and biases in clinician practice. Future studies will be necessary to further examine gender differences in critically ill patients. 

##  Key Messages

- In this cohort of older patients admitted to a medical intensive care unit (MICU), women were older than men and had more chronic respiratory disease but had otherwise similar baseline characteristics.
- Men and women in this older cohort received equivalent care in the MICU based on the Therapeutic Intervention Scoring System-28 (TISS-28).
- After adjusting for age and severity of illness, there was no difference in MICU or 15-month mortality.
- Contrary to prior studies in cardiac ICUs and mixed ICUs, there was no evidence of gender disparity impacting the amount of critical care delivered to this cohort of older patients.

## Figures and Tables

**Table 1:** ICU admission characteristics of patients enrolled in study  (*N*=309)*.

Characteristic	Men (*n* = 145)	Women (*n* = 164)	*P* value
Age in years, Mean (SD)	73.7 (8.2)	75.6 (8.6)	.046
Nonwhite race	19 (13)	32 (20)	.13
Medicaid status	13 (9)	30 (18)	.02
APACHE II Score	23.1 (6.6)	23.8 (6.2)	.36
Admitted from home	117 (81)	124 (76)	.28

Baseline Medical Status			

Body Mass Index (BMI, m^2^/kg), Mean (SD)^†^	25.7 (6.9)	26.1 (9.8)	.88
Chronic respiratory failure	37 (25)	64 (39)	.01
Chronic heart failure	33 (23)	44 (27)	.41
Chronic renal insufficiency	28 (19)	28 (17)	.61
Hepatic Failure	1 (<1)	1 (<1)	1.0
Malignancy	10 (7)	8 (5)	.45
Depression	31 (21)	54 (33)	.02
Dementia^†^	42 (29)	53 (33)	.55
Antidepressants prior to ICU admission	28 (19)	49 (30)	.03
Any Impairment in Activities of Daily Living	50 (34)	61 (37)	.62
Any Impairment in Activities of Instrumental Daily Living	120 (83)	144 (88)	.21

Full Code on ICU Admission^†^	121 (83)	144 (88)	.27
Admitting Diagnosis			

Sepsis	23 (16)	28 (17)	.77
Respiratory	68 (47)	88 (54)	.23
Neurologic	1 (<1)	4 (2)	.38
Gastrointestinal hemorrhage	33 (23)	19 (12)	.009
Other	20 (14)	25 (15)	.72

*All variables presented as *n* (%) except where indicated. ^†^Missing data present for some subjects. For BMI missing = 9; Dementia missing = 3; Code status missing = 1.

**Table 2:** Factors related to time in ICU (*N* = 309).

Continuous Descriptors of ICU Stay	Men (*n* = 145)	Women (*n* = 164)	*P* value*
	Median (IQR)	Median (IQR)	
Therapeutic Intervention Score (TISS-28) Range: 9–50	25 (14)	26 (14)	Equivalent at *P* ≤ .05****
Days of stay in ICU (LOS)** Range: 1–51	4 (5)	4.5 (4.5)	.38
Days of Intubation in ICU Range: 1–48	6 (8)	5 (6)	.69

	Men (*n* = 145)	Women (*n* = 164)	*P* value*
	*n* (%)	*n* (%)	

ICU Mortality	20 (14)	33 (20)	.14
ICU Delirium	110 (77)	129 (80)	.50
ICU readmission	13 (9)	16 (10)	.81
Intubation	78 (54)	89 (54)	.93
Use of vasopressor medications (Dopamine, dobutamine, norepinephrine, epinephrine, phenylephrine, vasopressin)	54 (17)	77 (25)	.0848
Tracheostomy	8 (5)	7 (4)	.61
Continuous Positive Airway Pressure (CPAP) or BiLevel Positive Airway Pressure (BiPAP)	30 (21)	42 (26)	.31
Pulmonary Artery Catheterization	10 (7)	19 (12)	.16
Enteral Nutrition (Nasogastric Tube or Percutaneous Endoscopic Gastrostomy Tube)	53 (37)	60 (37)	.99
Hemodialysis	7 (5)	10 (6)	.62
Continuous Veno-Venous Hemofiltration (CVVH)	5 (3)	8 (5)	.53
Change in code status to “Less aggressive”	37 (26)	45 (27)	.70

^†^Missing data present for some subjects. For ICU delirium missing = 5.

*Chi-square tests for categorical variables and Wilcoxon rank sum tests for continuous variables were used with statistical significance defined as *P* value ≤ .05.

**Length of stay was for first admission.

***Delirium was defined by either positive ICU Confusion Assessment Method (CAM) or chart indication of delirium during ICU stay.

****A nonparametric test of equivalence rejects the null hypothesis that men and women are not equivalent at significance level 0.05 for equivalence limits of ±15% around median value. IQR (Interquartile Range, i.e., central fifty percent distribution).

**Table 3:** Mortality and discharge disposition  (*N* = 309).

	Men (*n* = 145)	Women (*n* = 164)	*P* value
	*n* (%)	*n* (%)	
Death (ICU or Floor)	32 (22)	47 (29)	.17**^▲^**
Overall Mortality (15 months from ICU admission)	86 (59)	85 (52)	.76*
Discharge to skilled nursing facility, assisted living, rehabilitation unit, hospice, hospital**	54 (48)	67 (58)	.13**^▲^**

**^▲^**Chi-square or Fisher's exact tests were used for categorical variables with statistical significance defined as *P* value  ≤.05.

*The *P* value reported pertains to the interaction term in a Cox proportional hazards multivariable model including age, APS at admission, gender, TISS-28, and the interaction between gender and TISS-28.

**Those not discharged to Skilled Nursing Facility, and so forth were discharged to home or friend/relative.

Missing data are present for some subjects. For discharge location missing = 1.

## Abbreviations

ADL: Activities of Daily Living Scale
APACHE II: Acute Physiology and Chronic Health Evaluation
CCI: Charlson Comorbidity Index
COPD: Chronic Obstructive Pulmonary Disease
IQCODE: Informant Questionnaire on Cognitive Decline in the Elderly
IADL: Instrumental Activities of Daily Living Scale
ICU: Intensive care unit
SOFA: Sequential Organ Failure Assessment
SAPS: Simplified Acute Physiology Score
TISS-28: Therapeutic Intervention Scoring System-28
YNHH: Yale-New Haven Hospital.
